# Genetic Testing in Psychiatry for Individuals With Intellectual Disabilities: An Audit of Current Practice

**DOI:** 10.1192/bjo.2025.10695

**Published:** 2025-06-20

**Authors:** Megan Viegas, Sonya Rudra

**Affiliations:** South West London and St George’s NHS Mental Health Trust, London, United Kingdom

## Abstract

**Aims:** Individuals with intellectual disabilities (ID) have a higher prevalence of psychiatric conditions, that can be linked to underlying genetic syndromes. Identifying these conditions early can enable tailored treatment, informed prognostic counselling, and improved long-term outcomes. There are established criteria for genetic testing in individuals with unexplained moderate, severe or profound ID. This audit aimed to assess the proportion of eligible patients under a Community Learning Disability Team’s psychiatry service who had genetic testing discussed, referred, or completed.

**Methods:** A retrospective audit was conducted in a Community Learning Disability Community based in London. The electronic health records for all patients under the psychiatry caseload as of November 2024 were reviewed. Data extraction focused on the ID severity, details of genetic diagnoses and mention of clinical genetics testing within the notes. Specific search terms were used including “gene*”,” genome”, “congenital”, “test”, “investigation”, “diagnosis”, “karyotype”, “screen”, “chromosome”.

**Results:** Of the 94 patients reviewed,1 had profound ID, 16 had a severe ID, and 22 had a moderate ID. Among these individuals, 20.5% had a confirmed genetic diagnosis, including conditions such as Trisomy 21, Costello syndrome, and inherited glycosylphosphatidylinositol deficiency. Mentions of genetic testing – such as prior referrals, discussions, or test results – were found in 25.6% of patients with moderate or severe ID. However, only one patient had been referred for genetic testing within this team, with others being referred whilst under Paediatrics or Child and Adolescent Learning Disability teams.

**Conclusion:** This audit highlights a gap in the discussion and referrals for genetic testing within the Community Learning Disability team. Given the prevalence of genetic conditions in this population, and the potential impact on mental and physical health and management strategies, increasing awareness and embedding genetic testing discussions into routine psychiatric assessments is needed. Future steps include providing targeted education for the Learning Disability Team on the importance of clinical genetics, sharing the referral protocol to the local Clinical Genetics team, and considering the addition of a prompt in initial assessments to ensure genetic testing is routinely considered. These measures will enhance early identification, optimise treatment approaches, and improve long-term outcomes for individuals with ID and co-occurring mental illness.

